# eStroop: Implementation, Standardization, and Systematic Comparison of a New Voice-Key Version of the Traditional Stroop Task

**DOI:** 10.3389/fpsyg.2021.663786

**Published:** 2021-05-31

**Authors:** Riccardo Brunetti, Allegra Indraccolo, Claudia Del Gatto, Benedetto Farina, Claudio Imperatori, Elena Fontana, Jacopo Penso, Rita B. Ardito, Mauro Adenzato

**Affiliations:** ^1^Department of Human Sciences, Cognitive and Clinical Psychology Laboratory, Università Europea di Roma, Rome, Italy; ^2^Department of Psychology, University of Turin, Turin, Italy; ^3^Department of Neuroscience “Rita Levi Montalcini,” University of Turin, Turin, Italy

**Keywords:** cognitive control, executive functions, interference, facilitation, attention

## Abstract

The Stroop effect is a well-documented phenomenon, demonstrating both interference and facilitation effects. Many versions of the Stroop task were created, according to the purposes of its applications, varying in numerous aspects. While many versions are developed to investigate the mechanisms of the effect itself, the Stroop effect is also considered a general measure of attention, inhibitory control, and executive functions. In this paper, we implement “eStroop”: a new digital version based on verbal responses, measuring the main processes involved in the traditional effect. eStroop features four categories of stimuli in four different colors: (1) geometrical shapes, (2) neutral words, (3) congruent words, and (4) incongruent words. The results of the administration to 307 University students confirm the Stroop effect and offer baseline data for future research and clinical testing. Direct comparisons with other recent versions of the task are discussed, offering insights into differences and similarities between different task variables.

## Introduction

The “Stroop effect,” named after John Ridley Stroop in the 1930s, is a robust and well-documented (see MacLeod, 1991 for a review) demonstration of interference between two different cognitive processes, namely, an automatic one (e.g., reading) and a controlled one (e.g., naming a color; Stroop, 1935; see also Schneider and Shiffrin, 1977). This effect has been extensively studied using the Stroop task, one of the most widely used tasks in cognitive psychology (Gazzaniga et al., 1998). The traditional version presented participants with different words, printed in different colors, and asked them to name out loud the ink color, ignoring the words themselves. The original test was composed of two cards: a color card and an incongruent color-word card (Stroop, 1935; Experiment 2). Five colors were used: red, blue, green, brown, and purple. Later, some studies introduced other conditions in which the word was a color name congruent with the ink color (the word “Red” written in red ink; congruent words condition—MacLeod, 1991; Tzelgov et al., 1992), or the word was a color-neutral word (e.g., “Cat,” “Rabbit”; neutral words condition—MacLeod, 1991; Tzelgov et al., 1992). In the congruent words condition, participants reported faster reaction times (RTs), when compared with the neutral words condition, where the words were color-unrelated, showing a facilitation (Dalrymple-Alford and Budayr, 1966; Posner and Snyder, 1975; Fagot et al., 2009). Conversely, in the incongruent words condition (e.g., the word “Red” written in blue ink), participants' RT was slower than when performing the task in the neutral words condition. The increasing time to perform the incongruent words condition, compared with the other ones, is referred to as “the Stroop interference effect” (as in Davidson et al., 2003; Moering et al., 2004). MacLeod and MacDonald (2000; see also Goldfarb and Henik, 2007) proposed that the conflict between the task and the word meaning generates the Stroop interference. The authors named it “informational conflict”: a conflict between the notion of color, activated by color naming, and a conflicting color concept, triggered off by the reading process. However, interference is also found with neutral words since all words activate reading processes and, even non-word stimuli, like letter strings, can interfere (Klein, 1964; Sharma and McKenna, 1998). Indeed, most interpretations have considered the Stroop interference effect in terms of a response competition between the reading response to the irrelevant dimension of the task (e.g., the word itself) and the color-naming process. It seems that we have the inability to focus on both the color and the word (Treisman, 1969) as we can rely on a single-response channel (Morton, 1969): Since reading is an automatic process, it simply dominates the control process, and it occupies the channel before the controlled color-naming process can occur (Dyer, 1973). Automatic processes, like reading, are fast and are usually the result of a learning process, their implementation does not overload attention and can occur involuntarily (see also Del Gatto et al., 2021). The more a process becomes automatic through practice, the greater the interference caused by the encounter with a less automatic process will be (Schneider and Shiffrin, 1977; MacLeod, 1991). In contrast, controlled processes are slow, require attention, and are under voluntary control (Schneider and Shiffrin, 1977; Cohen et al., 1990). When there is a conflict between these two processes (e.g., reading and naming), our cognitive load is increased: Carrying out these tasks (e.g., stopping automatic reading, identifying word color, and solving information conflict) eventually slows down the response, significantly increasing RTs (see also D'Ausilio et al., 2010; Delogu et al., 2019 for similar effects in other domains).

To sum up, the Stroop interference effect is strong evidence of competition between automatic, task-irrelevant cognitive processes and a controlled, intentional cognitive one.

Finally, according to some authors, also gender differences seem to be relevant in the understanding of the mechanisms that affect the participants' response speed to the Stroop task. Indeed, results from Baroun and Alansari (2006), and Mekarski et al. (1996), seem to suggest that gender is important: Women seem faster in the Stroop task compared to men (Mekarski et al., 1996; Baroun and Alansari, 2006). Conversely, another study suggested that “there are no sex differences in Stroop interference at any age” as MacLeod stated (1991, p. 184).

The Stroop effect is still under investigation in order to fully understand some of its underlying mechanisms (e.g., Augustinova et al., 2019; Hershman et al., 2020) by using customized digital versions of the task programmed specifically for each research purpose, giving rise to myriad versions of the task. However, over the decades, the effect has also been addressed as a part of a larger theoretical frame (MacLeod, 1992): It is considered a general measure of attention and executive functioning (Moering et al., 2004) such as the ability to inhibit cognitive interference (Uttl and Graf, 1997). Indeed, inhibitory control, one of the three core executive functions, could enable us to inhibit our prepotent response to words reading, during the incongruent condition (MacLeod, 1991; Diamond, 2013).

During the years, the Stroop task was used to measure other cognitive functions such as attention processing speed (Jensen and Rohwer, 1966) and its relationship with working memory (Kane and Engle, 2003). Studies show that these skills decay with age (see also Dulaney and Rogers, 1994; Ivnik et al., 1996; Davidson et al., 2003; Huang et al., 2020) and in dementia (Houx et al., 1993); thus, the Stroop task became a popular test for the evaluation of various clinical conditions (e.g., frontal lesions Vendrell et al., 1995, see also Alvarez and Emory, 2006; Jurado and Rosselli, 2007 for a review; Parkinson's disease Fera et al., 2007; depression Markela-Lerenc et al., 2006). Moreover, the Stroop task has been widely used for assessing attentional deficits in neurological and psychiatric patients (Abramczyk et al., 1983; Blenner, 1993; Buchanan et al., 1994; Drake et al., 1996; Adenzato et al., 2019). All of these applications of the Stroop task as a general measure of cognitive elasticity and control were based on standardized versions, usually the Victoria version (Regard, 1981) or the Golden (1978) version: Both of these versions are originally physical and based on printed cards. Some studies digitized them, but without an assessment of their equivalence to the original ones (e.g., Moniz et al., 2016). Efforts to create standardized digital equivalents to the card-based versions often were influenced by variations from the physical to the digital versions (e.g., the manual response modality compared with the verbal one seems to show less interference: Penner et al., 2012).

To date, excluding the original card-based version, there is no solid standardized version of the Stroop task with respect to either the materials of the test, the administration, or the scoring method. Indeed, several different Stroop task versions have been developed, usually to investigate the specifics of the effect itself, with variations in the color and number of the test items, the number of subtests, and the administration procedure (e.g., Comalli et al., 1962; Golden, 1978; Trenerry et al., 1989). While these variations are useful for the purposes of specific studies, they do not make the Stroop task useful for the assessment of attention and cognitive control (MacLeod, 1992; Uttl and Graf, 1997; Moering et al., 2004). We will now illustrate the main differences found in the various versions of the task in the literature, in order to design a new digital version of the Stroop task that may serve for the abovementioned general measures of cognitive functioning.

## Materials Variations

The Victoria version (Regard, 1981) is composed of three different conditions. In the first condition, color names are presented in black ink; the second consists of colored disks; in the third one, color names are presented with an incongruent ink color (e.g., the word “Red” written in blue ink). While in the first condition participants had to read the words as quickly as possible, in the second and third conditions, the participants' task is to name the color of disks and printed words, respectively. The Victoria version uses, differently from the original Stroop experiments (Stroop, 1935), four colors. Other versions used fewer colors, three as in the most common Golden version (Golden, 1978), as few as only two (Hershman and Henik, 2019), or more, as many as six (Lamers et al., 2010).

The task-irrelevant features of the stimuli have been very flexible. The single geometrical shapes used by Stroop himself (Stroop, 1935), used as a baseline to evaluate the speed of the color-naming process without any interference, have been sometimes substituted by the same-letter or symbol strings (e.g., “XXXXXX,” Augustinova et al., 2019), mixed-symbol or mixed-letter strings (e.g., “!#>!##,” “shshshsh,” Levin and Tzelgov, 2016; Kinoshita et al., 2017), or strings of geometrical shapes matching color words in length (e.g., “▴▴▴▴▴,” Redding and Gerjets, 1977). As for the words themselves, different degrees and types of non-color or color-associated words have been used: neutral-color words (e.g., “Balcony,” Augustinova et al., 2010), non-color words beginning with the same letter as color names and matched for length (e.g., “Boat” for blue, “Rut” for red, Redding and Gerjets, 1977), color-associated words (e.g., “Tomato” Augustinova et al., 2010) with different degrees of frequency of use (Levin and Tzelgov, 2016), and color names themselves.

## Administration Procedure and Response Modalities

Despite variations, the basic paradigm of the Stroop task does not change: An incidental, automatic, and frequent response (reading) needs to be inhibited to perform an infrequent one (color naming). In the Victoria (Regard, 1981) and Golden's versions 1978, the Stroop task involved a series of items printed on sheets of paper and clustered by condition (“Simultaneous blocked” version). While in the Victoria version the result is expressed in the total time necessary to complete all items in each condition, in Golden's version the score is calculated as the total number of the successfully completed items in each block (card) in a given amount of time, most probably due to the fact that the procedure could be done with a common stopwatch. Later on, single trial versions of the test were proposed (“Serial” versions, e.g., Dalrymple-Alford and Budayr, 1966; Sichel and Chandler, 1969): In these versions, the different types of stimuli are administered either in “Blocked” order (all items of a specific condition in a sequence) or in an “Unblocked” order (e.g., randomly mixing all conditions). Stimuli could be displayed individually either on single cards (Dalrymple-Alford and Budayr, 1966), tachistoscopically (Tecce and Dimartino, 1965; Dyer and Severance, 1973), or on a computer screen (Spieler et al., 1996). Serial random presentation allowed for a more precise measurement of each individual response and possible sequence effects: The original blocked condition version did not contemplate an item-by-item RT analysis. Kindt et al. (1996), as well as Salo et al. (2001), compared the “Simultaneous” and “Serial” versions of the Stroop task. Both studies showed that the Stroop interference was larger in the “Simultaneous” version than in the “Serial” one. Error rates, conversely, appeared higher in the “Serial” version compared with the “Simultaneous” one. Interestingly, Kindt et al. (1996) suggested that using different task versions highlights different attentional processes. Indeed, in another study (Ludwig et al., 2010), comparing a “Simultaneous” card version and a computerized “Serial” one, the authors suggested that the “Serial” version offers a “purer” measure of the ability to resist the automatic reading process (see also Spieler et al., 1996). On the other hand, the “Simultaneous” version includes the ability to resist interference caused by surrounding distracting stimuli: The cards used for these versions need to be progressively scanned by the participant, who has thus to perform the task while actively ignoring surrounding distractors, which seem to create another kind of interference. Thus, the “Simultaneous” format seems to require additional inhibition to avoid the effects of surrounding distractors, on top of the inhibition necessary to avoid reading the current item (Ludwig et al., 2010).

Other variants of the test have used different kinds of response modalities: For example, Tecce and Happ (1964) asked participants to sort cards according to a colored rectangle. The task was performed more slowly when the cards featured also an incongruent color name, than when they featured only the rectangles. Usually, however, the traditional response modality of the Stroop task is a vocal one (naming the color out loud, Stroop, 1935). Another common response modality is to ask participants to detect colors with a keypress response (Pritchatt, 1968). The author found that when the keys were marked with color names, responses to stimuli featuring color names were slower than responses to colored rectangles. However, when the keys were labeled with color patches, there was less difference in response time between the two different kinds of stimuli (rectangles and color names). Some convoluted mixes of the two modalities were also explored: Mayas, Fuentes, and Ballesteros' (Mayas et al., 2012) participants responded orally (naming the color), and the experimenter recorded the participants' responses manually by pressing a key on a keyboard.

## Digital Stroop

Tecce and Dimartino (1965) pioneered the development of a computerized version of the Stroop task, measuring RTs with a voice-onset-operated relay. Since Tecce and Dimartino's version, a large variety of studies have used computers (Keele, 1972; Ehri and Wilce, 1979; Carter et al., 1995; Henik, 1996; Kindt et al., 1996, 1997; Girelli et al., 2000; Nichelli et al., 2005; Most et al., 2007; Jongen and Jonkman, 2008; Wright et al., 2015) or virtual reality (Henry et al., 2012; Parsons and Barnett, 2018). These computerized versions not only have enhanced the ecological validity (Chaytor and Schmitter-Edgecombe, 2003; Parsons et al., 2013), but have also allowed the researchers to compare the different outcomes generated by using a vocal or a manual response modality (MacLeod, 1991; Sharma and McKenna, 1998; Balota et al., 2000; Linnman et al., 2006; De Marchis, 2013). MacLeod (1991) stated that a manual response reduces interference, in comparison with a vocal one. He interpreted this difference as a stimulus–response compatibility effect: Since the stimuli are written words, a vocal response is more interfering than a manual one (Klein, 1964; Redding and Gerjets, 1977; McClain, 1983; MacLeod, 1991; Penner et al., 2012).

Recently, a digitized Stroop task has also been included in some test batteries (Björngrim et al., 2019). Mueller and Piper (2014) have created the psychology experiment building language (PEBL) test battery, an open-source version of common tests, in which the digitized version of Stroop task is based on a manual response. In the last years, Björngrim et al. (2019), digitized a series of cognitive tests, including the Stroop task, aiming at comparing traditional and digital versions of them. They compared a traditional paper-and-pencil and a digitized form of the Victoria version (Regard, 1981). While the traditional version participant responses were given orally, their digitized version required again a manual response. This difference in the response modality resulted in a different interference effect, as already pointed out above (see also Klein, 1964; Redding and Gerjets, 1977; McClain, 1983; MacLeod, 1991; Sharma and McKenna, 1998; Penner et al., 2012). Moreover, in the paper-based version, the authors used the total time as a measure, while for the digitized version they recorded response time for each item and then calculated the average response time for correct responses.

Other comparisons between the traditional and computerized versions of the Stroop task have highlighted controversial results. Gualtieri and Johnson (2006) showed that the computerized version of the task is equivalent to the traditional one; however, they did not focus their attention on the specific processes involved in the interference they trigger and they did not make a comparison with the verbal response modality (and this may lead to different effects: see MacLeod, 1991). Another study digitized the Stroop task (Gur et al., 2001) with congruent and incongruent trials and a manual response: Participants had to press colored keys on a computer game pad. A direct comparison shows moderate correlations between the traditional and computerized measures.

These controversial results might be explained by the fact that almost all the studies that digitized the Stroop task customized it for the specific needs of their research (e.g., using specific images, shapes, administration procedures, etc.). To the best of our knowledge, each digital version that was tested either relied on a manual response or, when using a verbal response, did not aim at replacing the traditional version as a general attentional and cognitive control measure.

## Processes and Conflict Types

While the interpretation of the effect by Stroop himself was based on a conflict between a more familiar reading response and a less familiar color-naming response (Stroop, 1935), it is nowadays commonly accepted in the literature that the increase in response times reflects the presence of inhibitory processes (MacLeod, 1991; Nigg, 2000; see also Aron, 2007 for a different interpretation Miller and Cohen, 2001). However, Treisman and Fearnley (1969) contrasted the traditional Stroop's interpretation of the effect as a conflict between a familiar and a less familiar response (Stroop, 1935). In a specifically designed Stroop-like task, based on card sorting according to either colors or words, they found a relatively small interference when the sorting criterion was based solely on words or on colors. But when the sorting was based on a mixed color-word criterion, the interference was much larger. The lack of interference in the color or word criterion was interpreted in terms of different cognitive processes involved in processing colors and words. Thus, in the color sorting task, when comparing only ink colors, the word reading process could be switched off, rendering the processing of word meanings unnecessary. In contrast, when the sorting task was based on a mixed color-word criterion, task interference arose because both properties (i.e., words and colors) needed to be processed in order to perform the task-relevant evaluation; consequently, both processes are involved: The ink color and word meaning can interfere with each other. Hence, the specific interference that arises in the Stroop task is interpreted by Treisman and Fearnley as caused mainly by the response modality compatibility with the task-irrelevant word meaning: a kind of stimulus-response compatibility.

Klein (1964) investigated specifically the underlying processes involved in the effect. The author used cards (“Simultaneous blocked”) with six different word types: non-sense syllables, rare words, common words, words semantically associated with colors (like “grass” and “sky”), color names from a set of colors different than those being named, and the standard incongruent color-name trials. Interestingly, the results showed different patterns of interference based on word types: shorter RTs for non-sense syllables until gradually reaching longer RTs for the incongruent condition.

In general, when we face two separate demands in a task, even when one is voluntary and the other is automatically triggered, a task conflict may emerge. It has been suggested that the Stroop task can generate two kinds of conflicts (Kalanthroff et al., 2013; Hershman and Henik, 2019): the task conflict (between the color-naming request and the automatic reading—that we will call *process interference*) and the information conflict (between the correct response and the word meaning—that we will call *semantic overlap*). The *process interference* between the color-naming task and the task-irrelevant word reading affects participants in all kinds of conditions including a word, since the word stimuli activate a spontaneous tendency to read (also see MacLeod and MacDonald, 2000; Levin and Tzelgov, 2016). The *semantic overlap* emerges in incongruent and congruent conditions because of the information similarity between word meaning and the color in which it is displayed (that represents the correct response to be given). Incongruent and congruent words conditions trigger both *process interference* and *semantic overlap*, with the key difference that in the congruent condition the *semantic overlap* becomes a facilitation process, while in the incongruent one, it becomes interference. When using neutral words, *process interference* is present, while *semantic overlap* is not; finally, in the case of stimuli not including words (e.g., letter or symbol strings, geometrical shapes), neither *process interference* nor *semantic overlap* is triggered. For this reason, the RTs of the conditions not including words are used as a baseline (Goldfarb and Henik, 2007; Kinoshita et al., 2017).

The above-mentioned literature makes evident that while the Stroop effect and the underlying processes are clear and well-known, the tools used to study this effect are miscellaneous, and they might produce some confusion. The eStroop, thus, emerges from the need to reduce this miscellany of the versions of the task.

## eStroop

Given the countless amount of Stroop task versions and the great variance between them, the aim of this study is to propose a cross-platform digital freeware version of the Stroop task that can be widely used and shared in clinical and research applications (see also Brunetti et al., 2014, 2018, for an example of research and clinical applications of eCorsi, a standardized digital version of the Corsi block tapping test), when the Stroop task is used as a general measure for attention and cognitive control. While a certain amount of customization is present in the program (e.g., the possibility to administer the task in different languages), eStroop does not aim to become a tool to specifically explore the mechanisms underlying the effect: Any research willing to investigate the variations of the processes involved in the Stroop task will certainly benefit from versions programmed *ad-hoc*.

**Figure 1:**
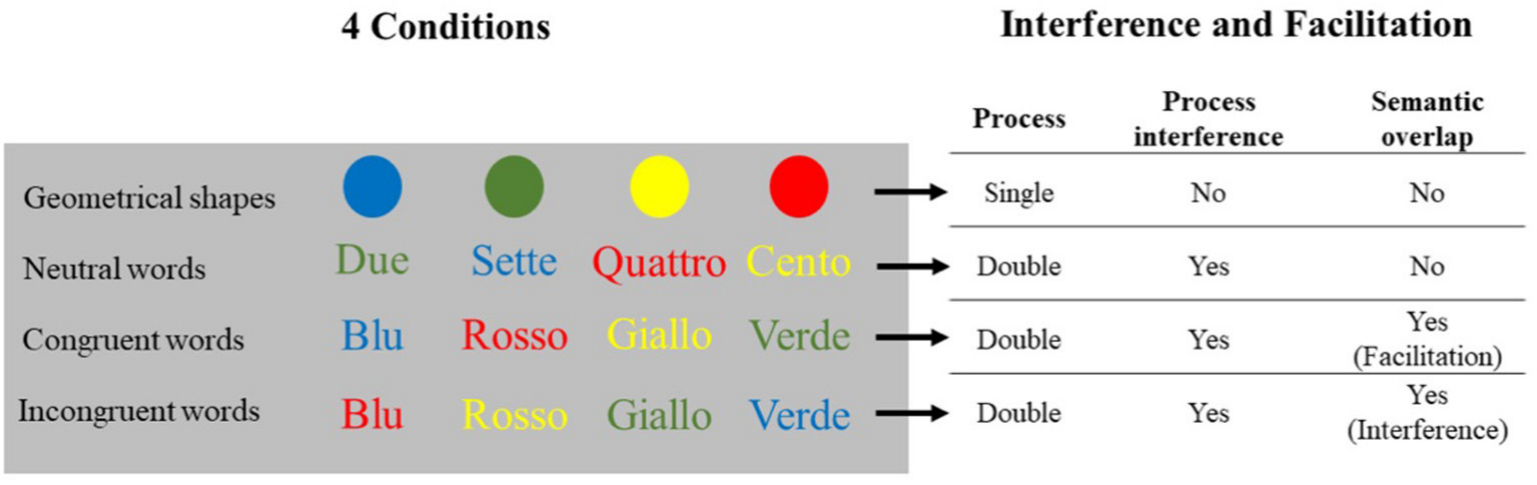
Graphic representation of the four categories of stimuli coupled with the specific interferences and facilitations.

This study describes the reliability and assessment of the eStroop task, along with a 2-fold purpose: The first is to overcome some limits present in the previous digital versions. As we have seen in the literature, in many cases digital versions are developed following the specific need of the research or involving the “Simultaneous” version, which has shown to be less accurate than the “Serial” one, or involving a motor responses modality (keypress) instead of a vocal one (which has been shown to trigger different effects in terms of interference, Sharma and McKenna, 1998; Repovs, 2004). We used a voice response mode and a “Serial unblocked” version, in order to obtain more reliable RTs and to single out each process involved (Ludwig et al., 2010). The second purpose is to offer a clear measure of the different patterns of interferences underlying the Stroop effect (e.g., Klein, 1964; Pritchatt, 1968), standardizing our contribution with a large sample of participants. To this purpose, we implemented four different conditions, using four types of stimuli: (1) geometrical shapes (disks), (2) neutral words (number names), (3) congruent words (congruent color names; e.g., “Red” written in red), and (4) incongruent words (incongruent color names; e.g., “Red” written in blue). Our hypothesis is to find different types of interference according to the conditions, specifically: (a) presenting “geometrical shapes,” we expect to obtain the fastest RT and the lowest error rate, since neither *process interference* (e.g., reading is not involved) nor *semantic overlap* will be present (single process); (b) “neutral words” will feature slower RTs (compared with the shapes) and some errors, since *process interference* will be triggered (MacLeod and MacDonald, 2000; Goldfarb and Henik, 2007; dual process); (c) “congruent words” will trigger both *process interference* and *semantic overlap*, showing a facilitation effect from the latter, with faster RTs and lower errors rate than in the neutral words condition, but still slower than geometrical shapes condition (congruent dual process); (d) “incongruent words” will trigger both *process interference* and *semantic overlap*, the latter this time actually slowing down the task, featuring the slowest RTs and the higher error rates (incongruent dual process, see Figure 1). Finally, our study explores possible gender differences and evaluates if the gender effect found with card versions arises also with our digitized version.

Moreover, since the Stroop task has been performed in many different ways, and since we are trying to create a tool that can be widely used, we will compare our results with different recent studies that implemented the task in a variety of ways (Levin and Tzelgov, 2016; Kinoshita et al., 2017; Augustinova et al., 2019). This comparison will help us to investigate potential differences and equivalencies between eStroop and other recent digital versions using verbal responses, to verify the reliability of eStroop results.

Kinoshita et al.'s 2017 aim was to compare the manual and the vocal response modalities in a Stroop task to investigate the different levels of interference that those may trigger. They used a “Serial unblocked” paradigm, featuring five different types of color-neutral distractors (real words, pseudowords, letter strings, symbol strings, and a string of Xs) as well as incongruent color words. Results show a different pattern of interference in the vocal and manual response modalities (e.g., slower RTs with the vocal task).

Levin and Tzelgov (2016) investigated the semantic gradient pattern and the distinction between two types of conflict—the task and the informational conflict involved in the Stroop interference. In their experimental investigation, performed both in Hebrew and in Russian, they used different conditions: incongruent words; high-frequency color-associated words; low-frequency color-associated words; high-frequency neutral words; low-frequency neutral words; letter strings; and geometrical shapes. They used a vocal response modality and a “Serial unblocked” paradigm. Results show the contribution of task conflict (orthographic component) and of informational conflict (direct informational conflict component) to Stroop interference offering substantiation that each type of conflict has its roles.

Augustinova et al.'s 2019 study examined whether *semantic overlap* and *process interference* are affected by the type of response output (verbal vs. manual). The authors used an extended form of the Stroop paradigm (Augustinova et al., 2018) with a “Serial unblocked” paradigm, and their results show a larger interference with the vocal compared with the manual modality, confirming previous studies (e.g., Kinoshita et al., 2017). Specifically, they showed that the response modality effect is due to a significantly lesser contribution of *process interference* to the overall Stroop effect when manual output, as opposed to vocal, is required. The stimuli they used in Experiment 1 consisted in color words, color-associated words, neutral words, and strings of Xs. In Experiment 2, in addition to the stimuli used in Experiment 1, they also used congruent stimuli in the form of color names and color-associated words.

## Method

### Participants

A total of 307 university students (71 males, mean age = 21.08 years; SD = 2.32 years; range = 18–33) took part in the experiment for course credit. The following inclusion criterion was considered: age between 18 and 35 years. Exclusion criteria were as follows: left-handedness; head trauma; diagnosis or a history of major psychiatric disorders; history of neurological diseases; and the previous central nervous system active drugs intake in the last 2 weeks before the assessment. A checklist with dichotomous items was used to assess inclusion/exclusion criteria and socio-demographic data. Moreover, all of them reported normal or corrected-to-normal vision; none of the participants suffered from color blindness. Power analysis was based on Aschenbrenner and Balota's 2019 results. Assuming a moderate effect size (Cohen's D ranging from 0.45 to 0.65) and using an alpha of.05 with the standard power of 90%, the sample size for the present study was a minimum of 36 (Cohen, 1988). The participants were naïve as to the purpose of the study and gave written consent in accord with the declaration of Helsinki; the experimental protocol has been approved by the Ethics Review Board of the Università Europea di Roma.

### Materials

We used four different stimuli categories in four different colors (red—RGB value: 255 0 0, blue—RGB value: 0 0 255, green—RGB value: 0 255 0, yellow—RGB value: 255 255 0) presented on a gray (55%; RGB value: 140 140 140) background: (1) geometrical shapes: disks (visual angle 2° 51′); (2) neutral words: number names (Italian: “Sette,” “Due,” “Cento,” “Quattro”; meaning for “Seven,” “Two,” “One hundred,” and “Four”; horizontal visual angle ranging from 4° 17′ to 7° 50′ according to word length, vertical visual angle 2° 35′; the words were chosen for their length similar to the Italian color names: “Rosso,” “Blu,” “Verde,” “Giallo”); (3) congruent words: color names congruent with the color they were presented in (e.g., the Italian word “Rosso,” meaning “Red,” presented in a red color; horizontal visual angle ranging from 3° 34′ to 6° 26′ according to word length, vertical visual angle 2° 35′); (4) incongruent words: color names incongruent with the color they were presented in (e.g., the Italian word “Rosso,” standing for “Red,” presented in a green color; same visual angles as the preceding condition).

### Procedure

The participants were seated in a quiet testing room, facing a monitor placed ~60 cm in front of their head. First, the researcher adjusts the microphone sensitivity to adapt it to the participant's tone of voice. Subsequently, the participants read the instruction on the computer screen and were invited to name out loud the color of the stimulus as fast as they can, ignoring the stimulus identity. This task was the same throughout the whole experiment, which lasted ~20 min. After receiving on-screen instructions, the participants performed 20 training trials featuring instances of all conditions, to get used to the task. Each trial began with a fixation cross randomly varying in duration (average = 2,000 ± 1000 ms) to avoid entrainment effects. Each stimulus lasted for 1,500 ms and in any case disappeared after the participants responded. Each condition had 40 instances, 10 repetitions for each color, for a total of 160 trials. The 160 trials were divided into four blocks of 40 trials each. Each block featured a random sequence of all stimulus categories (“Serial unblocked” paradigm), and at the end of each block, participants were invited to take a short break. All participants' responses were digitally recorded in sound files, and the RT was obtained using a voice key (vocal response). The sound files are then corrected thanks to a specific correction module in the program, allowing a human judge to filter out sound artifacts and to score hesitations (e.g., “mmmmm… red”) or corrections (e.g., “bl… red”) as incorrect responses, as all these cases, may have triggered the voice key with a false RT.

### Apparatus

The study was carried out using a laptop computer (MacBook Pro 15′). The computer was running “eStroop,” a custom-made script in Max 8 (Cycling ′74). The verbal responses and the voice key were, respectively, recorded and triggered using the laptop internal microphone.

## Results

Data analysis was conducted with SPSS 26 statistical package. Data on RT were tested for normality. Shapiro–Wilk test showed that data were normally distributed *W*_(71)_ = 0.99, *p* = 0.877, and as skewness (0.073) and kurtosis (−0.092) for male; and *W*_(236)_ = 0.99, *p* = 0.107, as skewness (0.125) and kurtosis (−0.375) for female. We ran a 2 × 4 mixed design analysis of variance (ANOVA) with Gender (2, between factor) and Condition (4, within factor) as independent variables. Using RTs as a dependent variable, the analysis yielded a significant main effect of Condition [*F*_(3, 915)_ = 348.261, *p* < 0.001, partial *η*^2^ = 0.533], while the main effect of gender and the interaction was not significant [respectively: *F*_(1, 305)_ = 0.149, *p* = 0.700 and *F*_(3, 915)_ = 0.275, *p* = 0.844]. *Post-hoc* analysis (Bonferroni) on Condition revealed significant differences between all four categories (all comparisons *p* < 0.001; Table 1).

**Table 1:** Means (standard deviations) of reaction time (RT) and percentage of accuracy of the four conditions.

	**Neutral words**	**Congruent words**	**Incongruent words**	**Symbols/** **Shapes**
RT (ms)	717 (113)	676 (101)	772 (116)	635 (85)
Accuracy (%)	98.91 (3.1)	99.3 (3)	97.3 (3.8)	99 (3.7)

Data on Accuracy were tested for normality. Shapiro–Wilk test showed a significant departure from normality both for male and for female groups, respectively, *W*_(71)_ = 0.740, *p* < 0.001, and *W*_(236)_ = 0.338, *p* < 0.001. Thus, we ran the Kruskal–Wallis non-parametric test on Accuracy that yielded no significant results.

### Systematic Comparison Results

**Figure 2:**
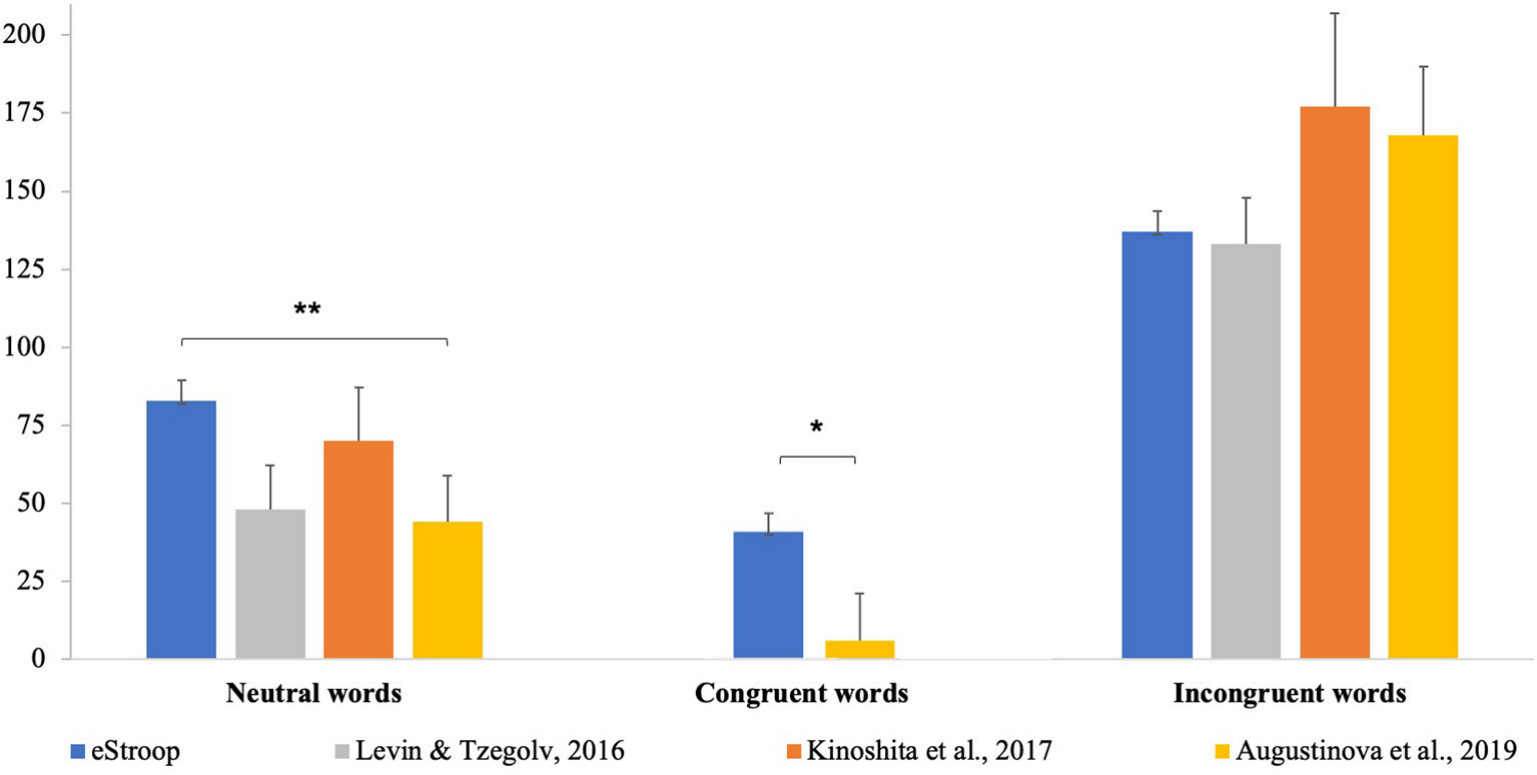
Chart of delta values in ms (the subtraction of mean values of the shapes/strings conditions from the neutral, congruent, and incongruent conditions in all studies). *, significant differences ≤0.05; **, significant differences ≤0.01.

We compared RT data from the vocal response conditions of Augustinova et al. (2019), Kinoshita et al. (2017), and Levin and Tzelgov (2016), with our data (see Table 2 for a summary of all RTs results). We calculated the difference in RTs by subtracting a non-readable baseline (e.g., shapes, letter string, or similar, see Table 3) from the congruent, incongruent, and neutral words conditions of all studies (see Figure 2). We chose to calculate this difference to focus our comparison on the specific interference effects found in each study, avoiding possible discrepancies due to methodological differences between them. We have thus selected the following conditions:

- Kinoshita et al. (2017): Their “real-word” condition (words unassociated with colors) became the neutral words condition chosen for the comparison, along with their incongruent words condition. The baseline condition we subtracted to the neutral and incongruent words conditions is their “XXX” condition (string of Xs).
- Levin and Tzelgov (2016): From Experiment 1, their “neutral high frequency” condition (NeuH) became the neutral words condition chosen for the comparison, along with their “incongruent color words” (CW) that became the incongruent words condition. The baseline condition we subtracted to the neutral and incongruent words conditions is their “geometric shapes” condition.
- Augustinova et al. (2019): From Experiment 2, their “color neutral words” condition became the neutral words condition, their “standard-color congruent words” became the congruent words condition, and their “standard-color incongruent words” condition became the incongruent words condition were chosen for the comparison. The baseline condition we subtracted to the neutral, congruent, and incongruent words conditions is their “Strings of Xs” condition.

**Table 2:** Means RT (standard deviations) of our study and the other study we compared.

	**Neutral words**	**Congruent words**	**Incongruent words**	**Symbols/** **Shapes**
eStroop	717 (113)	676 (101)	772 (116)	635 (85)
Kinoshita et al. (2017)	651 (87)	n.p.	758 (94)	581 (74)
Levin and Tzelgov (2016)	674 (73)	n.p.	759 (78)	626 (63)
Augustinova et al. (2019)	695 (130)	645 (130)	819 (191)	651 (113)

*n.p., data not present*.

**Table 3:** Delta values (standard deviations) calculated by subtracting mean RT of neutral words, congruent words, and incongruent words to the baseline (symbols/shapes).

	**Δ** ** Neutral—baseline**	**Δ** ** Congruent—baseline**	**Δ** ** Incongruent—baseline**
eStroop	82 (113)	41 (101)	137 (116)
Kinoshita et al. (2017)	70 (87)	n.p.	177 (94)
Levin and Tzelgov (2016)	48 (73)	n.p.	133 (78)
Augustinova et al. (2019)	44 (130)	6 (130)	168 (191)

*n.p., data not present*.

We ran three different ANOVAs, one for each condition (neutral, congruent, and incongruent), comparing our delta data with the deltas calculated from Kinoshita et al. (2017), Levin and Tzelgov (2016), and (Augustinova et al., 2019, see Table 3).

ANOVA results for the **neutral words delta** showed a significant main effect [*F*_(3, 429)_ = 2.752, *p* = 0.042]. Tukey's honestly significant difference (HSD) *post-hoc* significant results:

eStroop vs. Augustinova et al. (2019): *p* = 0.04

ANOVA results for the **congruent words delta** showed a significant main effect [*F*_(1, 382)_ = 6.478, *p* = 0.011]. Tukey's HSD *post-hoc* significant result:

eStroop vs. (Augustinova et al., 2019, vocal): *p* < 0.001

ANOVA results for the **incongruent words delta** did not show a significant effect [*F*_(3, 429)_ = 1.658, *p* = 0.17].

## Discussion

The results of the experimentation with eStroop showed, as expected, that the “geometrical shapes” condition (single process) obtained the fastest RTs, as shown by the *post-hoc* comparison, and could be used as baseline for the other conditions (double process): This is due to the fact that in this condition, the reading process is not triggered and the response is the result of a single process. In the “neutral words” condition, featuring color-unrelated words, obtained longer RTs, showing the presence of *process interference*. This follows the interpretation of the “neutral words” condition as triggering a double process (the task-relevant color-naming and the task-irrelevant reading response) and consequently creating the interference. The “congruent words” condition featured RTs significantly longer than the “geometrical shapes” condition, yet shorter than the “neutral words” condition. The task in this condition features again a double process (triggering a *process interference*), therefore resulting more demanding than the condition where only a single process is activated, but, interestingly, there is a significant advantage compared with the “neutral words” condition. This advantage is due to a congruency effect between the response required by the task and the contents of the automatically read word: The *semantic overlap* generated takes thus the form of a facilitation. In other words, when more processes are present, we observe a *process interference* that slows down responses (as in the “neutral words” condition), but thanks to the *semantic overlap*, the content congruency of the two processes creates a facilitation, speeding up the responses (as in the “congruent words” condition). Lastly, as shown by the *post-hoc* results, in the “incongruent words” condition, we found the longest RTs compared with all the other conditions. This is a result of the joint effect of both *process interference* (e.g., a double process is triggered) and *semantic overlap* (e.g., the contents of the task-relevant process are similar to the contents of the task-irrelevant process), the latter resulting in interference caused by information incongruency. These results are in line with previous literature (MacLeod and MacDonald, 2000; Hershman and Henik, 2019), showing that the largest interference in terms of RTs seems to emerge due to a conflict both in terms of process and in terms of semantics. In our study, both the *process interference* and the *semantic overlap* appear to be clearly discernable, with specific joint effects, as rendered evident from the comparison of our four conditions (e.g., the effect of *process interference* seems to add to the *semantic overlap* in the incongruent condition, while the *semantic overlap* partially cancels out the *process interference* in the congruent condition).

Investigating gender effect, differently from other studies (Mekarski et al., 1996; Baroun and Alansari, 2006), our results showed no gender difference neither in general response speed (main gender effect absent) nor in the specific amount of interference (no significant interactions), generally confirming the absence of sex differences (MacLeod, 1991).

From the comparison with other studies, results show that both in the “neutral words” condition and in the “congruent words” condition, our effect is significantly different from Augustinova et al. (2019). Specifically, the comparative analysis of congruent words conditions showed that eStroop generated larger RTs than Augustinova et al.'s 2019 results. This difference can be explained by the different array of conditions used in the two studies. Augustinova et al. used multiple congruency levels (standard color-congruent and associated color-congruent words), while in our study only a single level of congruency was included. Augustinova et al.'s study thus included a significantly larger number of congruent trials (while eStroop featured 40 congruent trials, their study had a total of 96 congruent trials), which can result in a training effect, as pointed out also by the original Stroop's study 1935. A similar result was obtained in the “neutral words” condition: Again, eStroop *process interference* appears to be significantly larger than the one obtained by Augustinova et al. (2019). We can speculate that this difference is due to the specific neutral stimuli used by Augustinova and colleagues: Their “neutral” words are common words, such as “dog.” While common words are not associated with specific colors (e.g., a “dog” can feature different colors), a potential bias in association with color may still be present: A “dog” may be hardly associated with some colors such as green, purple, or blue, as it is not possible to find dogs of those colors in nature. This limitation may, unfortunately, be relevant for any word representing something concrete. Our use of abstract concepts as numbers, while still at risk of personal color associations, minimizes such potential biases. Such color association biases may create an unwanted *semantic overlap* additional effect also in the “neutral words” condition, thus not showing a pure *process interference* effect. The difference between our results and Augustinova et al.'s results 2019 in the “neutral words” condition may be an effect due to a different amount of *semantic overlap* influence between the two studies, triggered by the different types of words used. Another possible explanation of these discrepancies between the two studies could be due to the different baseline conditions chosen (geometrical shapes in our study and a string of Xs in Augustinova et al.): A difference in these conditions, since we used them as a baseline, would affect the amount of interference in the other conditions. However, this latter cause should elicit a similar effect also in comparison with Kinoshita et al.'s study 2017, since we also chose their string of Xs condition as baseline. Since this last difference is not significant, the first explanation seems more plausible.

In the “incongruent words” condition, we did not find a significant difference between our results and the other studies. eStroop results are therefore similar to other digital Stroop versions featuring a vocal response, showing that eStroop is a valid and reliable tool that is able to replicate their outcome. Unfortunately, a direct comparison with the traditional “Simultaneous” version (which are still the most used ones) is not possible, as the scoring methods of the Victoria (Regard, 1981) and the Golden's 1978 versions are inherently based on cumulative RTs, while the “Serial” versions are based on a single-trial analysis.

As we have seen, eStroop gives the possibility to analyze in detail the different types of mechanisms underlying the Stroop effect: *process interference* and *semantic overlap* (as pointed out since Klein, 1964, and confirmed by Dyer, 1973; MacLeod and MacDonald, 2000; Goldfarb and Henik, 2007). A close study of the differences between these two mechanisms can lead to a better understanding of an effect that has been intriguing psychologists for almost 100 years.

Although our study demonstrates that eStroop results are reliable and similar to those obtained by previous versions, we can detect some limitations: First, our sample is not heterogeneous, including only undergraduate students, with a larger number of women. Moreover, our sample only includes healthy participants, while it would be crucial to perform a systematic analysis on the performance of different clinical populations. Second, we did not compare the gender, age, and educational status of our sample with the sample used by other studies. Third, we did not include IQ as a variable in our study: This is a relevant limitation since several authors suggested that performance based on executive functions (e.g., cognitive inhibition) is related to IQ scores (Ardila et al., 2000; Friedman et al., 2006). Fourth, there was no explicit validity analysis (e.g., theoretical, criterion) as the Stroop effect is already a valid effect, with considerable literature analyzing its validity. Fifth, this study does not provide any data about reliability, as it was not performed with a design allowing for a reliability analysis (e.g., test–retest). Sixth, at least some of the differences identified in our comparison with other studies may be due to the different number of trials administered, or to the peculiarity of the stimuli used in each study. Finally, the task was administered in Italian and not in English or in other languages, limiting its results. eStroop, however, is easily customizable, allowing an easy translation of its stimuli in any other language.

## Conclusions

In this paper, we tested, standardized, and offered proof of equivalency with previous similar versions, of a new tool for psychological assessment: eStroop, a freeware, cross-platform digitized version of the traditional Stroop task. When compared with other versions, eStroop shows several advantages, including a standardized set of easily translatable stimuli capable of highlighting the main processes involved, a valid voice key to measure RTs with millisecond precision, and a customizable administration design. This study provides standard data, obtained with single trial analyses, from a large sample of young participants (university students). All these features show that eStroop is not only useful for diagnostic purposes, but it can come in handy in the field of research, as it allows the analysis of controlled and automatic mechanisms sometimes difficult to be investigated with traditional “Simultaneous blocked” methods or with other digital versions. Moreover, it offers a free, reliable tool for all the research wishing to use a standardized digital Stroop task^1^, without wishing to change radically its features. eStroop allows for a direct comparison and robust meta-analyses of results from different populations, a comparison now easier to perform without any fluctuation due to different stimuli, different procedures, or different response types. Finally, we believe that eStroop is more accessible and user-friendly than other automated or digitized versions, as it is based on technology which is nowadays widely available.

## Data Availability Statement

The raw data supporting the conclusions of this article will be made available by the authors, without undue reservation.

## Ethics Statement

The studies involving human participants were reviewed and approved by Ethics Committee, Università Europea di Roma. The patients/participants provided their written informed consent to participate in this study.

## Author Contributions

RB designed the interface. RB, AI, and CD produced the initial hypotheses, analyzed data, and drafted a first version of the manuscript. BF, CI, EF, and JP were responsible for administration and initial data processing. RA, MA, BF, RB, AI, and CD reviewed and revised the final manuscript. All authors contributed to the article and approved the submitted version.

## Conflict of Interest

The authors declare that the research was conducted in the absence of any commercial or financial relationships that could be construed as a potential conflict of interest.

## Footnotes

^1^All materials and the *eStroop* administration package are accessible by asking the corresponding author.
